# How Do Social and Behavioral Change Interventions Respond to Social Norms to Improve Women’s Diets in Low- and Middle-Income Countries? A Scoping Review

**DOI:** 10.1016/j.cdnut.2024.103772

**Published:** 2024-05-11

**Authors:** Kate Litvin, Gargi W Grandner, Erica Phillips, Lisa Sherburne, Hope C Craig, Kieu Anh Phan, Avni N Patel, Katherine L Dickin

**Affiliations:** 1 USAID Advancing Nutrition, John Snow, Inc., Arlington, VA; 2 Nutritional Sciences, Cornell University, Ithaca, NY; 3 Nutritional Sciences, University of Wisconsin – Madison, Madison, WI; 4 Public and Ecosystem Health, Cornell University, Ithaca, NY

**Keywords:** social and behavior change, nutrition, gender norms, women’s dietary diversity, health systems, food systems

## Abstract

Healthy dietary practices are highly influenced by social norms, the widely-held expectations about the behaviors that are appropriate or typical within a given group. However, many nutrition programs designed to reduce women’s undernutrition in low- and middle-income countries do not address the influence of social and gender norms in their interventions, and therefore, there is limited information about how norms-responsive interventions have been designed and implemented. The objective of this scoping review was to identify and describe social and behavioral change interventions designed to improve women’s dietary practices and nutritional intake that integrate the influence of social and gender norms. We systematically searched 4 databases (Scopus, Web of Science, PubMed, and CINAHL) for peer-reviewed articles describing design, implementation, and/or assessment of nutrition interventions in low- or middle-income countries. Results are reported following Preferred Reporting Items for Systematic Reviews and Meta-Analyses guidelines. Our review identified 27 articles from 25 projects or research studies that addressed social or gender norms related to women’s dietary practices. The majority focused on the pregnancy and lactation periods, and a few aimed to reach all women of reproductive age. Interventions most often endeavored to shift norms through multiple activities, channels, and platforms, aiming to reach not only the primary participants but also influencers and reference groups. Intervention approaches ranged from home visits and support groups to engage influential family members to community-level outreach with opinion leaders such as religious leaders, health care workers, and peer change agents. Most interventions were delivered through the health sector or were community-based, with some nutrition-sensitive agriculture interventions. There is increasing, although still limited, integration of social and gender norms perspectives in the design, implementation, and assessment of interventions to improve women’s diets. This comprehensive review summarizes influential norms and intervention approaches, an important step toward enhancing the effectiveness of social and behavioral change interventions by addressing nutrition-relevant norms.

This study was registered at Open Science Framework as JSBF7.

## Introduction

Improving women’s nutrition is central to ending hunger and achieving food security, the second United Nations Sustainable Development Goal (SDG) [1]. Progress on meeting this goal and improving women’s nutrition more broadly has been inadequate in many low- and middle-income counties (LMICs) [2,3]. More than 1 billion women and girls around the world do not have access to healthy diets, and micronutrient deficiencies among adolescents and women remain unacceptably high [4]; in some countries, >80% of women have inadequate intake of key nutrients [5]. Neither SDG 2 nor the WHO Global Nutrition target for maternal nutrition will be met by 2025 if current trends continue [2,6].

Beyond promoting improved practices to individuals, ensuring that women have healthy diets requires addressing the broader contextual barriers and facilitators that influence the uptake of recommended behaviors. Although financial resources and food security (or lack thereof) have long been recognized as determinants of an individual’s dietary intake, social and cultural factors related to gender roles and women’s status in households, communities, and societies, such as family support, are increasingly considered in health and nutrition social and behavioral change (SBC) interventions in LMICs [[7], [8], [9], [10], [11]]. Social norms are an example of broad, communal factors that play an important role in influencing health and nutrition behaviors [[12], [13], [14], [15], [16]]. Social norms are the perceived informal, spoken or unspoken, rules about what behaviors are appropriate, typical, or obligatory within a given group and reflect the influence of family and community beliefs on individual behavior [17]. An individual’s behavior can be driven by one’s beliefs about what most people do (descriptive norm) and perceptions about what others might approve or disapprove of (injunctive norm) [18]. Norms are upheld and reinforced by influencers or reference groups, i.e., “relevant others whose behavior and (dis)approval matter in sustaining the norm” [19].

Norms influence women’s dietary practices and nutritional outcomes in multiple ways, including shaping cultural and social expectations of what is appropriate or typical regarding women’s access to food and resources and allocation of time for nutrition-relevant roles [16,20]. Social norms operating within the household context can influence how family food resources and needs are translated into practices that shape women’s diets, thereby affecting their nutritional status, health, and well-being. Norms may support or constrain uptake of recommended evidence-based nutrition behaviors. For example, the expectation that women be provided with nutritious diets or “special” foods during pregnancy or postpartum is a positive norm. Perceptions that women should sacrifice their own nutrition for the sake of other family members create norms that negatively influence women’s diets. When a woman’s status is low, the combination of restricted power relative to other family members and harmful social norms can create critical barriers to improving nutrition, even if food access, affordability, nutrition knowledge, and skills are addressed.

Programs promoting practices to prevent women’s undernutrition often concentrate on the first 1000 d, the time from conception through a child’s second birthday, due to the immediate effects of maternal nutrition on birth outcomes. However, an exclusive focus on pregnant and lactating women fails to address the intergenerational impacts of undernutrition [21,22], the need to improve nutrition prior to conception, and women’s involvement in feeding, nurturing, and providing for children aged >2 y [7,23,24]. Beyond this, a policy and program focused only on maternal diets neglects women’s own lifetime health and well-being outside the first 1000 d and their right to adequate food, distinct from their roles as mothers [24].

The importance of norms has been increasingly recognized in some areas of global health such as reproductive health and family planning interventions [24,25]. However, the role of social and gender norms in shaping the uptake and sustainability of improved dietary practices in interventions is often overlooked [26]. A 2018 review article [11] about barriers to maternal nutrition in the first 1000 d included social norms as one potential barrier but did not focus on strategies to reduce these barriers or the design or implementation of interventions.

Our interest in conducting this review was to explore if and how SBC interventions that aim to improve women’s diets and nutritional intake have been designed to address relevant social norms. To our knowledge, this is the first review of this topic. Identifying and summarizing this information through a scoping review highlights relevant approaches and strategies, identifies implications and gaps for research, and provides information to support further integration of social norms into interventions to enhance program effectiveness.

### Objectives of the review

We conducted a scoping review to comprehensively summarize how interventions in LMICs have sought to address social norms to improve women’s diets. We used search terms to include all women of reproductive age, regardless of pregnancy or lactation status, because the negative effects of undernutrition throughout the reproductive years can have detrimental consequences for women, children, and their families. We considered norms related to dietary practices and food intake, including dietary diversity, diet quality, and quantity of food intake, along with select nutritional outcomes, including iron-deficiency anemia, BMI, and gestational weight gain. The objectives of this review were to a) identify interventions that addressed social and/or gender norms to positively influence women’s diets and dietary intake in their design, implementation, and/or assessment phase, b) describe how norms were included in these interventions, including the types of norms, stated theory of change and/or pathways of influence, and delivery characteristics, and c) identify implications and gaps for intervention approaches and research.

## Methods

We followed a systematic approach based on the Joanna Briggs Institute’s methodology [27] and the PRISMA Extension for Scoping Reviews reporting guidelines [28]. We identified and reviewed quantitative, qualitative, and mixed-methods studies that report the design, implementation, and/or assessment of interventions with ≥1 norms-focused behavior change component intended to improve women’s diet-related behaviors, as described below. The protocol was registered at Open Science Framework (https://osf.io/jsbf7).

### Inclusion and exclusion criteria

We determined eligibility for inclusion in this review based on the Peters et al. (2015) [27] framework of population, concept, context, and studies.

Population: Women aged between 15 and 49 y, including all stages of pregnancy and lactation or broader samples that included analysis specific to women within this age range.

Concept: Interventions that addressed social and/or gender norms influencing diet-related behaviors, with the intention to reduce women’s undernutrition. These could include health, nutrition, gender, and/or nutrition-sensitive interventions.

Context: Countries consistently defined as low- or lower-middle-income economy based on World Bank Income Classification for fiscal years during the period of the review [29].

Studies: Full-text articles were included if they were published between January 1990 and August 2023 and described relevant interventions, were peer-reviewed, and written in English. Articles were excluded if they focused exclusively on outcomes related to women’s overnutrition or micronutrient supplementation, were review articles, were descriptive research not linked to an intervention, or reported change in social norms as an outcome without an intervention intentionally designed to build on or influence them.

### Literature search strategy

We searched 4 databases: Scopus, Web of Science, PubMed, and CINAHL on 31 August, 2023. Search terms were from 5 domains matching the criteria described above, including the following: *1*) women of reproductive age; *2*) dietary intake and assessment; *3*) social and gender norms; *4*) behavioral interventions; and *5*) LMIC contexts (Supplement 1).These domains were developed by consensus based on the review objectives, consulting relevant literature, and expertise of the authors in previous work on social norms [16,25,30].

A team of 8 reviewers conducted title/abstract screening and full-text review using Covidence Online Software [31]. Two reviewers independently screened each abstract and reviewed full-text articles using checklist tools developed for ensuring consistency in the study selection process. Conflicts or uncertainties at each stage were resolved by a third voter or group consensus.

### Data extraction and synthesis of results

Six reviewers independently extracted data using a pretested extraction form created in Covidence. If multiple articles from the same study or intervention were identified, data were extracted and reported together.

## Results

### Summary of identified articles

**FIGURE 1:**
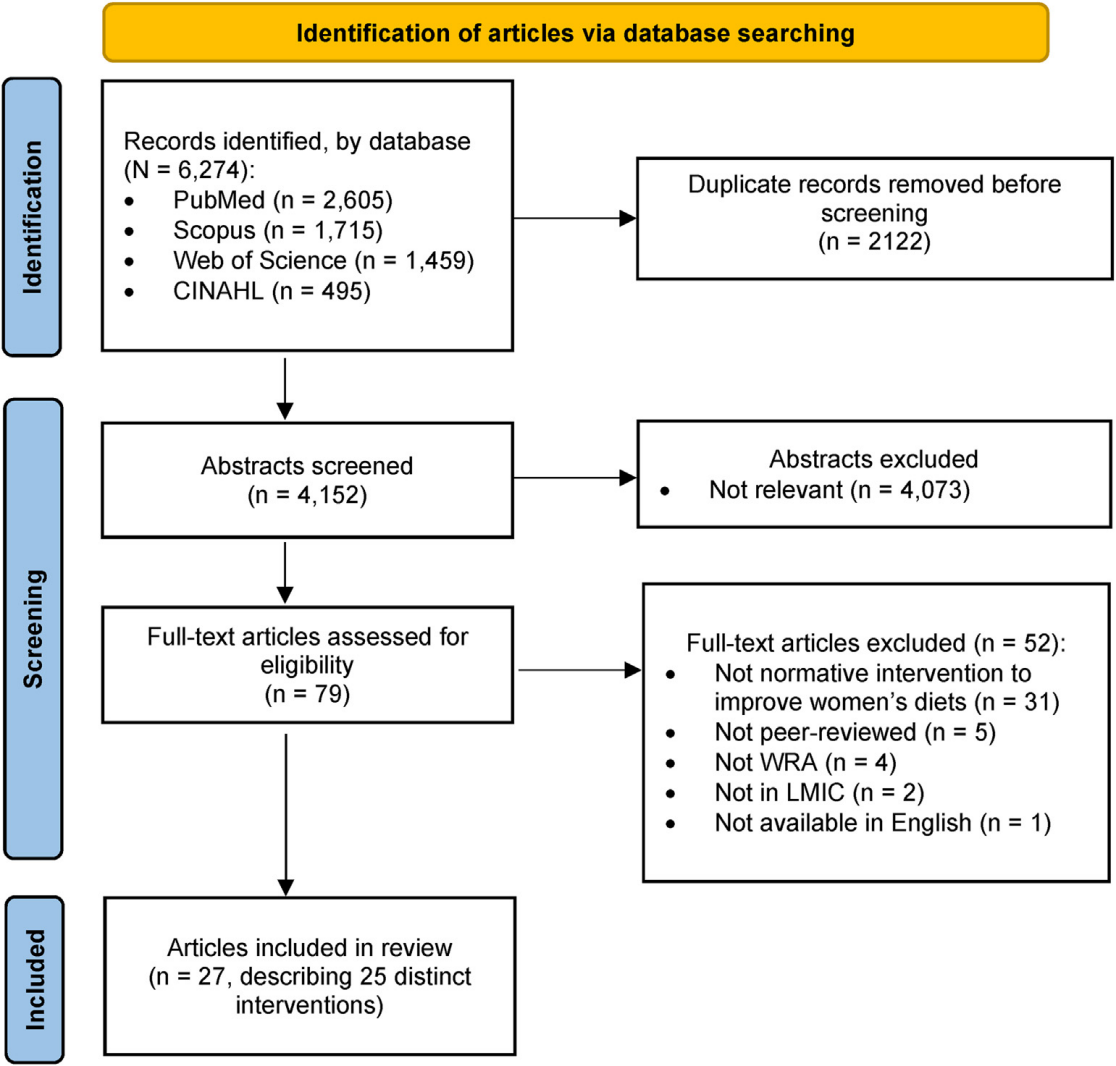
Flow chart of search and screening results of scoping review of interventions to improve practices related to women’s diet and social and gender norms. LMIC, low- and middle-income country; WRA, women of reproductive age.

We screened 6274 abstracts and identified 27 articles from 25 distinct projects or studies describing intervention approaches related to social norms and women’s dietary practices (Figure 1). Some articles used the terms “social norms” or “gender norms,” whereas others described norms using phrases such as “cultural beliefs” or “food-related beliefs.” Articles that met the screening criteria described in the protocol were included, regardless of the authors’ terminology. The 25 projects included intervention approaches designed and/or implemented in sub-Saharan Africa (*n* = 9) and Asia (*n* = 16), including 11 in South Asia and 5 in East and Southeast Asia. No articles were identified between 1990 and 1999, 2 articles (7%) were published between 2000 and 2009, 10 (37%) were published between 2010 and 2019, and 14 (52%) between 2019 and 2023.

We summarized the range of social norms addressed in the articles by grouping norms into 2 categories: food-focused social norms and gender norms (Table 1) [[32], [33], [34], [35], [36], [37], [38], [39], [40], [41], [42], [43], [44], [45], [46], [47], [48], [49], [50], [51], [52], [53], [54], [55], [56], [57]]. These categories overlap, as all norms related to women’s diets may have a gendered element, by definition, with some directly related to food and others more related to expectations around women’s and men’s roles or food distribution within the household. Food-focused norms included those related to food selection or choice, food quantity, and perceived value of foods. Gender norms includes those related to women’s roles and household decision making, intrahousehold food allocation, and ideals and expectations related to gender. Only one project targeted urban communities only; 16 were conducted in rural areas and 8 in both rural and urban areas (Table 2) [[32], [33], [34], [35], [36], [37], [38], [40], [41], [42], [43], [44], [45], [46], [47], [48], [49], [50], [51], [52], [53], [54], [55], [56], [57], [58], [59]]. Almost all interventions were multipronged, with multiple household and community activities targeted to women and at least one reference group or influencer; these were most often delivered through the health sector (Table 3) [[32], [33], [34], [35], [36], [37], [38], [39], [40], [41], [42], [43], [44], [45], [46], [47], [48], [49], [50], [51], [52], [53], [54], [55], [56], [57],^59^].

**TABLE 1:** Summary of social norms relevant to interventions to improve women’s diets

Norms addressed, by category		Articles (*n*)	Social norms or perceptions of norms and expectations	Reference group or influencers	List of articles (Author, year)
Food-focused norms (*n* = 18)	Food selection/ choice	16	• Most people expect pregnant women to avoid eating certain foods to avoid harm to the infant. • Specific foods women are expected to eat in pregnancy or lactation. • Most people expect postpartum women to avoid raw and “cold” foods including fruits and vegetables and eat more “hot” foods. • Some people expect pregnant to eat have priority/eat high-value foods during pregnancy (not breastfeeding).	Husbands, mothers-in-law or other female elders; group leaders, health workers, lay counselors, community health workers, government officials/policy makers	Alam 2020 [32]; Aubel 2004 [33]; Bao 2010 [34]; Cole 2016 [35]; Entsieh 2015 [36]; Galvin 2023 [37] Kadiyala 2016 [38]; Khani Jeihooni, 2021[39]; Liu 2006 [40]; Morrison 2021 [41]; Nandi 2014 [42]; Nguyen 2017 [43]; Ochieng 2017 [44]; Shivalli 2015 [45]; Yilma 2020 [46]; Talegawkar 2021 [47]
	Food quantity	5	• Mothers-in-law and other family members expect pregnant women to eat less to avoid complications during delivery and harm to the infant.	Grandmothers, mothers-in-law, other female elders	Aubel 2004 [33]; MacDonald 2020 [48]; Dykes 2012 [49]; Nandi 2014 [42]; Shivalli 2015 [45]
	Value of foods	3	• Most people value imported fruits and expensive food over locally available food including the fruit and traditional vegetables. • Women expected to sacrifice own nutritional needs by prioritizing other family members’ share of nutritious and valued foods.	Family, mothers-in-law, agricultural extension agents, community health workers, health workers	Alam 2020 [32]; Cole 2016 [35]; Ochieng 2017[44]
Gender norms (*n* = 15)	Women’s roles and household decision-making	10	• Mothers are responsible for nutrition, but men decide if certain types of food are present or absent in the household. • Most husbands in the village do not purchase diversified nutritious foods and ensure that their wife has these foods available. • Mother-in-laws and other family members expect women to continue working throughout pregnancy.	Husbands; grandmothers, mothers-in-law; older family members; CHW, lay counselors, volunteers (men or mothers)	Aubel 2004 [33]; Entsieh 2015 [36]; Isler 2020 [50]; Morrison 2021 [41]; Nandi 2014 [42]; Nguyen 2017 [43]; Nguyen 2018 [51]; Prost 2022 [52]; Ragasa 2021[53]; Ridolfi 2019 [54]; van den Bold 2015 [55]
	Intrahousehold food allocation	8	• Mothers-in-law and other family members prioritize food for men who work in the fields and expect women to do the same. • Mothers must ensure that everyone in the household receives enough food, sometimes at her own expense. • Most women expect to share the nutritious food with the whole family and sacrifice for others, in pregnancy and preconception. • Mothers-in-law and/or husbands determine amount and timing of meals.	Husbands, grandmothers, mothers-in-law, other female elders, co-wives, community health workers	Alam 2020 [32]; Diamond-Smith 2022 [56]; Galvin 2023 [37]; Isler 2020 [50]; Morrison 2021[41]; Nandi 2014 [42]; Ragasa, 2021 [53]; Yilma 2020 [46]; Talegawkar 2021 [47]
	Ideals and expectations related to gender	1	• Women prioritize feeding family members, not their own diets. • Most people do not expect women to take initiative to ensure a diverse diet for themselves during pregnancy	Not applicable; these are internalized or personal norms	Katenga-Kaunda 2020 [57]

Abbreviation: CHW, community health worker.

**TABLE 2:** Description of intervention characteristics and approaches related to social norms

Lead author, year	Intervention name	Program context	Theory/rationale	Intervention approaches	Intervention scale and duration	Social norms addressed	Priority behaviors
Alam 2020 [32]	Balanced Plate Intervention	Sherpur District, Bangladesh; rural communities	None noted	Monthly home visits to provide antenatal care services, including nutrition education with demonstrations for all family members by trained CHWs (Shasthya Kormis).	893 individuals in 36 rural clusters, 25 women each, 3 mo postpartum	Intrahousehold food distribution Food-focused norms: what women should eat during pregnancy; value of locally available food (fruits, fish).	Pregnant women eat a diverse diet. Pregnant women increase in food intake.
Aubel 2004 [33]	Grandmother Project	Thiadaye and Joal Districts, Senegal; rural communities	Health promotion, community psychology and adult education. Grandmother Inclusive Approach based on a family systems theory.	4 nutrition education sessions.	13 communities, 9 mo	How much women should continue working during pregnancy. Food-focused norms: what pregnant women should eat.	Pregnant women eat a diverse diet including iron-rich and “special foods” provided by grandmothers; increase food intake. Pregnant women reduce workload.
Bao 2010 [34]	Diet and Lifestyle Intervention Study in Postpartum Women	Shandon, Hubei and Guangdong Provinces, China; semiurban and rural hospitals and health centers	Social Ecological Model	One antenatal care counseling session, with brochures and take-home videos, and 4 postpartum counseling visits. Counseling hotline. Postpartum workshops for father and grandmothers. Hung posters for community members and engaged heads of health centers.	Plan for 800 women, with 4 data collections postpartum and follow-up to 2 y	What postpartum women should eat to recover from childbirth. Sitting month: lie in bed all day without physical activity to maintain energy balance.	Postpartum women eat a diverse diet, including fruits, vegetables, and milk. Postpartum women adopt a more active lifestyle.
Cole 2016 [35]	Mama SASHA (Sweet Potato Action for Security and Health in Africa)	Bungoma and Busia Counties, Kenya; catchment communities of 8 rural health facilities	Program theory to prepare program impact pathway	Community clubs for PLW, and monthly dialogs including cooking demonstrations. Health workers counseled pregnant and lactating women on diet and provided 2 vouchers for vines.	8 health facilities (4 program/4 control), 3 y	Low value of foods such as orange-fleshed sweet potato; seen as “women’s crop” and “poor person’s food” and “sick person’s food.”	Pregnant women grow and eat vitamin-A rich orange-fleshed sweet potato.
Diamond-Smith 2022 [56]	*Sumadhur (*“Best Relationship”)	Nawalparasi, rural District of Nepal	None stated	Weekly group intervention for wives, husbands, and mothers-in-law that included nutrition, anemia, intrahousehold food allocation, gender inequitable norms and practices, and couples and household relationship dynamics.	Formative work included 200 participants Pilot intervention 90 people (30 triads), 4 mo	Expectations of women’s role and intrahousehold allocation of food.	Newly married women do not eat last and have access to nutritious foods.
Dykes 2012 [49]	Nutrition Support Programme	Nahaqi, Khyber Pakhtunkhwa, Pakistan; communities surrounding and served by the Emergency Satellite Hospital with Pakistanis and Afghan refugees	Context-Mechanism-Outcome framework to design the research prior to the intervention	Weekly cooking demonstrations with nutrition education and food “incentives” (milk powder, rice, pulses). Nutrient supplements for malnourished women. Community nutrition education including men.	Communities surrounding the hospital, 12 mo; formative research with 16 health workers	What women should eat during pregnancy and lactation.	Pregnant and lactating women increase intake of food. Pregnant and lactating women eat a diverse diet.
Entsieh 2015 [36]	Mobile Midwife	4 regions in Ghana; rural communities; Qualitative study conducted in Awutu Senya District	Health belief model	mHealth messages in local languages to pregnant women and new parents.	4 regions, on-going since 2010; focus groups conducted with 29 pregnant or nursing mothers	Pregnancy and food preparation are women's responsibilities. Foods that women should avoid eating during pregnancy.	Pregnant and lactating women eat a diverse diet. Fathers support pregnant women in food preparation and other tasks.
Galvin 2023 [37]	Engaging Fathers for Effective Child Nutrition and Development in Tanzania (EFFECTS)	Mara Region, Tanzania, rural communities	Theory of change designed by researchers and implementors	Peer group sessions; due to COVID-19, pivoted to home visits for the last 6 mo	80 villages, 960 households; (Data collected from 815 mothers and 733 fathers at endline); 12 mo	Social and gender norms around parenting, and household communication and decision making, including dietary diversity.	Mothers consume a diverse diet; fathers involved in parenting and promotion of gender-equitable attitudes.
Isler 2020 [50]	Mobile Nutrition Video-Based Intervention	Nouna region, Burkina Faso; urban and rural communities	Gender framework for the research	Home visits to pregnant and breastfeeding mothers to show a set of maternal nutrition videos on a tablet.	1 region, 70 qualitative data collection activities	Intrahousehold food allocation; roles of women and men in food access and preparation.	Pregnant and lactating women eat a diverse diet.
Kadiyala 2016 [38]	Pilot to Integrate nutrition BCC into Agricultural Extension	Keonjhar District, Odisha, India; rural communities with women's self-help groups	None stated	10 videos for existing women’s self-help groups with facilitated discussion.	30 villages, 10 mo	What food pregnant and lactating women should eat.	Pregnant and lactating women eat a diverse diet.
Katenga-Kaunda 2020 [57]	Nutrition Education for Pregnant Women in Rural Malawi Cluster-RCT	Mangochi District, Malawi; rural areas that rely on subsistence fishing and/or farming	Theory of Planned Behavior	Nutrition education and cooking demonstrations monthly with powders of fish, vegetables, and legumes. Weekly individual counseling sessions by lay counselors.	257 women from 20 communities, 12 mo	Subjective norms: women not seeing own need for diet diversity in pregnancy as a priority.	Pregnant women eat a diverse diet.
Khani Jeihooni 2021 [58]	Quasi-experimental study to reduce anemia in pregnancy	Shiraz City, Iran; urban health centers	Theory of Planned Behavior	6 education sessions that included presentation of materials and group discussion. One session included husbands and health center officials.	142 pregnant women in 2 health centers, 6 wk	Pregnant women’s perception of husbands’ and health staff’s support of nutrition behaviors to reduce anemia.	Pregnant women should consume iron-rich foods.
Liu 2009 [40]	Health and Nutrition Education Intervention	Hubei, China; urban and rural areas served by hospitals and health centers	None stated	2 antenatal care counseling sessions, and 4 postpartum counseling visits.	410 participants, 11 mo	What foods women should avoid eating postpartum to recover from childbirth. Sitting month: stay inside with closed windows; avoid bathing.	Postpartum women eat a diverse diet, including fruits. Postpartum women adopt a more active lifestyle/ leaving the house.
MacDonald 2020 [48]	Mamanieva Project	Bonthe District, Sierra Leone; rural communities	Grandmother Inclusive Approach based on a family systems theory	Peer groups of grandmothers, community dialogs and intergenerational fora using songs, games, picture discussion cards, and stories-without-an-ending. Community-wide days of praise of grandmothers.	15 communities, 4 y	How much food women should eat during third trimester in pregnancy.	Pregnant women increase intake by one additional meal a day. Lactating mothers increase food and liquid.
Morrison 2021 [41]	Anemia intervention (formative research)	Kapilvastu district, Province 5, rural plains of Nepal	Conceptual framework adapted from Bronfenbrenner’s (1977) ecological model. Tan empowering education approach informed by Paulo Freire’s Theory of Education	Home visits with pregnant woman (4–16 wk apart in mid-pregnancy), including grandmothers and men. “Collective” group intervention using a 14-mo PLA cycle to identify the problem and develop locally appropriate response strategies; led by female CHVs once per month, includes pregnant women, grandmothers, men, and community members.	16 women, 20 fathers, 19 mothers-in-law	Power and positionality, gender norms, trust in health services and harmful norms at the individual, family and community levels.	Pregnant women eat a micronutrient rich diet.
Nandi 2014 [42]	Mitanin Programme	Chhattisgarh State, India; rural communities	Program theory and vision for change that defined health as encompassing social determinants	Family level outreach with demonstrations, community-organization building and social mobilization on health, along with health system advocacy.	All rural hamlets of the state, 60,000 CHWs, 1 y	Roles and status of women, including use of violence and withholding food. Denial of food to women immediately postpartum.	Pregnant and lactating women increase intake of food.
Nguyen 2017 [43]	Maternal, Newborn and Child Health Program by BRAC and Alive & Thrive	14 districts, Bangladesh; urban and rural communities	Conceptual framework of factors influencing maternal nutrition practices	Home visits by CHW to provide nutrition education monthly.	14 districts, 24.9 million mothers and children, on-going monthly since 2010	Influence of husbands and older family members in women's nutrition. What foods and how much food women should eat during pregnancy.	Pregnant women eat a diverse diet.
Nguyen 2018 [51]; Wable Grandner, 2022 [59]	Nutrition-focused Maternal, Newborn and Child Health Program by Alive & Thrive	10 subdistricts from 4 districts, Bangladesh; urban and rural communities	Theory of Reasoned Action	Home visits by 2 CHWs monthly to deliver interpersonal counseling. Husbands’ forums, small group meetings with community opinion leaders, video shows, and theater for the community. Print materials for community leaders and family members.	2000 wives and 1514 husbands at baseline in 20 subdistricts in 4 districts, 1 y (Nguyen)	Husband’s role in maternal nutrition (Nguyen); CHW strategies to persuade influential family members to support recommendations (Wable Grandner).	Pregnant women increase intake of food. Pregnant women eat a diverse diet.
Ochieng 2018 [44]	Good Seed Initiative	Arusha region, Tanzania; urban and rural communities	Theory of change	Road shows, cook shows, and campaigns in hospitals, schools, markets and villages to improve diet diversity, and create demand for traditional vegetables and market incentives for producers.	1 region, 1 y	Value of traditional foods.	Women of reproductive age eat a diverse diet with traditional vegetables
Prost 2022 [52]	Upscaling Participatory Action and Videos for Agriculture and Nutrition (UPAVAN)	Odisha State, India; 4 rural administrative blocks in Keonjhar District	Theory of change, designed by researchers	A trial of 3 nutrition-sensitive agriculture interventions with participatory videos and women’s group meetings.	148 villages randomly assigned; 4291 women interviewed at the endline; 32 mo	Family and gender norms related to household decision making and “enabling environment.”	Pregnant women and mothers with children under 2 consume adequate diets.
Ragasa 2021 [53]	Nutrition and gender behavior change communication for dietary diversity	Central Dry Zone, Myanmar; Rural villages	None stated	Alternating group-level meetings with household- and individual-level coaching. This was adapted due to COVID-19 restrictions to household visits and coaching sessions, then phone-based coaching.	30 villages randomized; 918 households at baseline, 503 households at endline; 11 mo	Gender-influenced decision-making related to purchases, preparation and allocation and food.	Women consume grater dietary diversity; Empowerment related to decision making.
Ridolfi 2019 [54]	Family Farms for the Future	4 districts, Cambodia; rural and peri-urban farming households	Gender analysis framework	Participatory discussions with key family members using the adapted Nurturing Connections curriculum.	4500 households in 4 districts, 1 y	Family support and relationships with women; Women’s empowerment focused on decision-making related to assets.	Women of reproductive age eat a diverse diet.
Shivalli 2015 [45]	Trials of Improved Practices (TIPs) to Enhance the Dietary and Iron-Folate Intake during Pregnancy	Uttar Pradesh State, India; rural communities majority vegetarian	Trials of Improved Practices	2–3 home visits using interpersonal communication, with the active participation of family members and reminder print materials.	98 pregnant women, 4 communities,12 wk	What foods and how much food pregnant women are allowed by others to eat, including eggs and milk.	Pregnant women increase food intake. Pregnant women eat a diverse diet (including eggs, greens).
Talegawkar 2021 [47]; Yilma 2020 [46]	Reduction in Anemia through Normative Innovations (RANI)	Odisha, India; rural communities, primarily Hindu	Theory of Normative Social Behavior	Women's self-help groups meetings. 10 PLA sessions and community engagement meetings with displays of women’s hemoglobin counts. Engage health officials at multiple levels and policymakers at the state level.	3797 individuals in 89 village clusters,11 mo	Intrahousehold food allocation, what foods pregnant women are allowed by others to eat, especially iron-rich foods; Dietary diversity.	Pregnant women eat iron-rich foods, dietary diversity.
van den Bold 2015 [55]	Enhanced-Homestead Food Production (E-HFP) Programme	Gourma Province, Burkina Faso; rural communities	BCC strategy based on the agriculture to nutrition pathways	Village Model Farms, run by women Village Farm Leaders where women learned about homestead food production and small-animal rearing. Provided women with inputs for their own home production activities.	1757 men and women at baseline in 55 communities, 2 y	Women's control over and ownership of productive assets, including gardens to grow fruits and vegetables.	Mothers of young children eat a diverse diet.

Abbreviations: BCC, behavior change communication; CHV, community health volunteer; CHW, community health worker; PLA, participatory learning and action; PLW, pregnant and lactating women; RCT, randomized controlled trial.

**TABLE 3:**
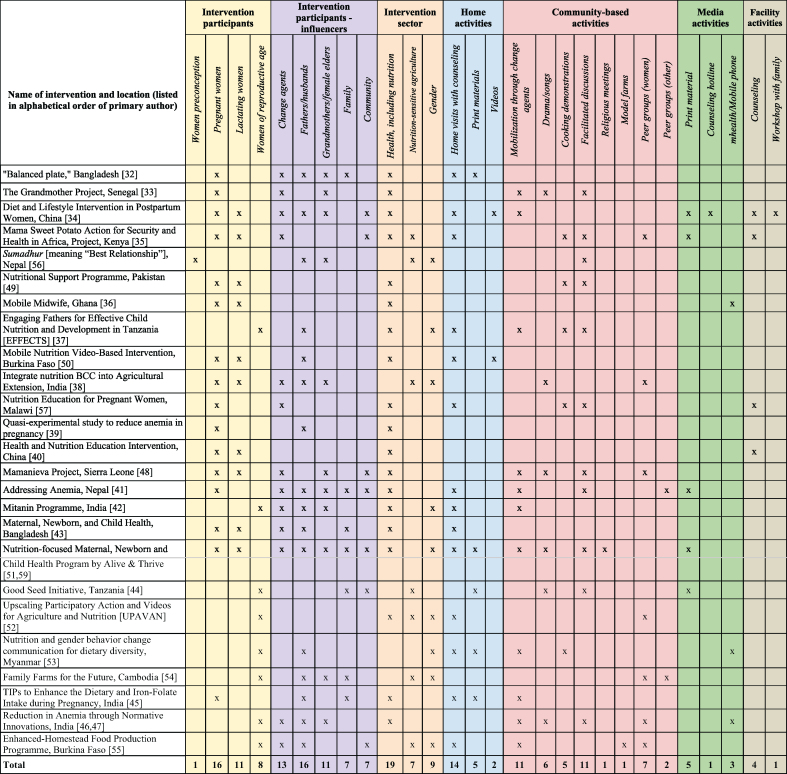
Summary of intervention characteristics

### Norms addressed in interventions to improve women’s diets

Seventeen articles described food-focused social norms, including norms that influence food choice/selection, food quantity, and/or value of foods. Of these, most articles (*n* = 16) referenced norms related to food choice or types of foods women are expected to eat or not eat, most often during pregnancy or while breastfeeding. For example, women’s consumption of certain foods during pregnancy, such as crabs and mangos in some contexts, was perceived to risk a large newborn and difficult delivery or harm to the infant [33,36,46,49]. Three other articles described hot and cold typologies of foods that influence what postpartum women are expected to consume or avoid [34,40,41]. Five articles addressed norms related to food quantity, such as expectations that women eat less during the last months of pregnancy to avoid a larger infant and potential complications during childbirth [33,42,45,48,49]. One article also described attempts to shift norms related to food quantity during lactation [49]. Three articles described norms related to the value of foods or types of foods perceived as appropriate for women. One found that imported, expensive produce was perceived as more desirable for pregnant women than locally available food [32].

Over half of the articles referred to gender norms that influenced women’s diets, including women’s roles, intrahousehold food allocation, and ideals and expectations related to gender. Nine articles described norms related to intrahousehold food allocation, including the expectation that women prioritize food for other family members, especially their husbands. A study in Nepal described the traditional practice of pregnant women eating last to ensure that food provided to the family remains “pure” [41]. In 3 articles, family members perceived men to require a greater quantity of food compared with women, due to physical size or demanding labor [33,42,56]. Only 1 article measured internalized norms, assessing how women’s perceived ability to prioritize their own healthy diets influenced adoption of new behaviors [57].

### Intervention design

We summarized features of the design and/or implemented normative interventions, any theories of change cited, and the dietary behaviors and practices that the intervention aimed to address (Table 2). All studies sought to impact ≥1 relevant behaviors by addressing norms that influence women’s dietary diversity (*n* = 21) or adequate intake of food and dietary adequacy (*n* = 9). Some interventions also aimed to improve specific nutritional outcomes, such as improving weight gain during pregnancy (*n* = 2), reducing underweight (*n* = 1), increasing women’s BMI (*n* = 1), and reducing micronutrient deficiencies through dietary intake (*n* = 6).

All except 4 interventions referred to a program framework, theory, or conceptual model. Ten of the interventions were based on SBC or systems theories, such as the theory of normative social behavior, family systems theory, and the socioecological model. Eleven interventions applied program frameworks, including theories of change and program impact pathways that included the influence of norms as a determinant on behavioral intention. Articles published since 2020 were more likely to include descriptions of programmatic pathways or a theory of change developed by the researchers following formative research.

To identify sociocultural beliefs and practices, 16 of the 27 articles in our review described conducting formative research. Most used focus group discussions and/or in-depth interviews, and other methods included participatory activities (i.e., drawings, vignettes, voting, transect walks), trials of improved practices, and surveys. Thirteen articles included results of formative research and 10 described how these findings influenced intervention design. For example, formative research helped identify family and community members who were influential in defining and enforcing norms [33,41,48], food customs for women [35,41], and how social and gender norms influence intrahousehold food allocation, perceptions of appropriate food for men and women, and household roles and responsibilities [50,54,56].

### Normative intervention approaches

Fifteen of the 25 projects were implemented exclusively within the health sector, including nutrition, and 2 were designed to influence the food system through nutrition-sensitive agriculture (Table 3). No project was exclusively gender-focused, but 9 projects included a combination of at least 2 of these 3 sectors. Almost all interventions (*n* = 17) specifically intended to reach pregnant women, the majority of which (*n* = 11) also intended to reach breastfeeding women. Eight projects aimed to reach women as mothers when their children were within a specific age range (e.g., <2 or <5 y), women as household decision makers (sometimes reaching married couples), or a combination of these. One project focused solely on the preconception period and one on nonpregnant women of reproductive age.

Fourteen projects included >1 intervention approach and engaged people at various levels: households and communities and through services and media. Eighteen interventions included a community component and 16 included a household component. Six intervened solely at the community level and 3 at the household level exclusively. Household-level approaches included home visits (*n* = 14) to women and their families by trained change agents; 2 interventions began engaging fathers during home visits after hearing this request from women during formative research [41,50]. An intervention that focused on women’s internalized norms used monthly nutrition education sessions and individual counseling during home visits to introduce powders of dried fish and legumes and enable women to add these nutritious powders in food prepared for the whole family, so benefits would be shared by all family members, including women [57].

More than half of the interventions equipped community leaders, health workers, agricultural extension agents, and community volunteers to advocate, mobilize, and implement activities to influence women’s roles, diets, and care. In these contexts, elders are respected and influential members of families and communities, so some interventions engaged older female relatives, including grandmothers, as advisors and caregivers (Table 3) [48]. Several intervention approaches included change agents who modeled new, positive norms. For example, Helen Keller International’s Enhanced-Homestead Food Production program in Burkina Faso worked with community leaders to contribute land to establish Village Model Farms run by female Village Farm Leaders who were also program participants [55]. This was a departure from most household-level model farm approaches, which tend to favor males in leadership and management roles.

Nine interventions included one-time or occasional community gatherings to reflect on existing and/or potential shifts in social norms related to nutrition, including women’s nutrition and diets. Most engaged the broader community in dialogs or public fora, but several engaged women and men separately, often with cooking demonstrations for women. One intervention conducted group education sessions and discussions between newly married women, their husbands, and mothers-in-law [56]. Some reinforced positive norms, such as encouraging intake of nutritious foods during pregnancy and lactation.

Six articles described integrating normative change efforts into women’s peer groups led by trained facilitators that met regularly to discuss and provide mutual support on nutrition and/or agriculture topics. For example, some interventions used a “collective” group approach involving women and other family and community members [41], added videos to existing women’s self-help groups for agriculture [38], and had a gender-based curriculum for multiple household members [54].

Interventions delivered through health services or media included efforts to shift food-based norms through counseling at the health facility during antenatal and postnatal care visits [34,40]. One intervention also used phone hotlines [34], and 3 interventions used mobile phone messages to shift norms, one doing so due to COVID-19 restrictions [36,46,53]. None used television, radio, or social media platforms.

It is worth noting that although authors described interventions designed to shift or build on social norms, there was wide variation in the assessment of norms and methods used to evaluate if or how norms changed over time. As this was a scoping review, summarizing data on effectiveness was not a key objective. To guide future work, however, it was important to consider the extent to which norms were measured. Fourteen of the 25 projects measured norms as a process or final outcome; however, only 9 assessed *changes* in social norms related to women’s dietary practices. Three of these used quantitative methods, assessing husbands’ perceptions of other husbands’ support for their pregnant wives [51] or asking women about perceived influence of family members on their ability to consume a diverse diet [56,57]. These studies found that perceptions of enabling social norms were associated with improvements in women’s dietary diversity or intrahousehold allocation.

Other projects used qualitative methods and reported improvements in support and engagement of husbands and mothers-in-law and intrahousehold food distribution [32], women’s access to food postdelivery [42], and increased consumption of healthy foods during pregnancy [36]. One project reported that recognizing and encouraging grandmothers and other elders to support women’s nutrition led to changes in community norms related to nutrition during pregnancy [33]. A program that promoted model farms with women farm leaders for homestead food production and small-animal rearing found that women had greater decision-making power and control over home gardens and their produce [55].

## Discussion

This scoping review identified 27 articles from 25 projects and research studies that reported intervention planning or implementation of approaches aimed at influencing social and gender norms to improve women’s diets, either by responding to norms that negatively influenced women’s diets (e.g., by shifting harmful norms) or by building positive norms. Within these 25 projects, there was a range of definitions, detail, and focus related to norms. The majority of interventions focused on women during pregnancy and lactation, less frequently targeting all women of reproductive age, despite the importance of women’s nutrition for their own and their families’ well-being. Most of the social norms that were discussed, even when linked to particular foods, reflected how family and community power structures and the status of women affected dietary intake. Recognizing the complexity of altering these systems, interventions most often aimed to shift norms through multiple activities, channels, and platforms, simultaneously directed toward primary participants, influencers, and reference groups.

Although most projects identified theoretical underpinnings of SBC, the majority did not specify how the intervention approaches were meant to shift social norms. Only 2 used the same theory (Theory of Planned Behavior) to design or assess their intervention [57,58]. Without clear theories and pathways, the quality and approach to assessment and evaluation of social norms was inconsistent among projects. The older evaluations in our review did not collect data on intermediate variables expected to mediate between the intervention and outcomes, nor on process indicators of fidelity and participation, whereas the more recent ones did, an indication of progress in this area.

Some authors acknowledged that norms are difficult to shift, and the implemented intervention approaches did not overcome traditional gender roles and food customs. For example, the authors of one article noted some supportive changes in normative beliefs but also found that some mothers-in-law still felt that traditional family and gender roles should guide intrahousehold allocation of food [32]. In another evaluation, women said they had no reason to disregard traditional food beliefs in favor of mHealth messages on dietary diversity during pregnancy, suggesting the mHealth messages alone were not sufficient to change perceptions [36]. Sometimes intervention effectiveness in shifting harmful norms was limited by the challenges of engaging key influencers and reference groups; one study that found improved nutrition knowledge and attitudes of women in postpartum did not translate to behavior change without more engagement of female elders [40].

Potential resistance to and unintended consequences of norms-shifting interventions should be considered in program planning, implementation, and assessment. Evidence indicates that women’s perceptions of supportive norms among family members is associated with improvements in women’s dietary practices [51,57]. The potential harm of advising women to eat better when they lack the family and community support to do so should be considered as part of program design (in consultation with community members) and monitored during implementation [9,60]. Because social norms are embedded within institutions, change may be needed at the level of laws and policies, as well as among communities and individuals, to effectively shift these “meta-norms” [30].

Women’s practices related to allocating nutritious or high-status food to certain household members may be influenced by social norms as well as other costs and benefits of favoring specific individuals in the household, depending on gender and life-cycle stage [61]. Only one article in our review identified internalized norms, pointing to a gap in knowledge about how interventions can address women’s personal norms about putting family before themselves or otherwise striving to be an ideal woman, wife, or mother. It is possible such norms are seen as too culture-bound or entrenched to address effectively within the scope of existing nutrition interventions [62]. Further participatory and experiential research is needed to explore possible strategies to surface and examine internalized norms, which may be most feasible in contexts where such norms are in transition or not held universally or where living situations permit women to make changes (i.e., female-headed or nuclear households).

We did not aim to analyze the effectiveness of norms-focused approaches or the role of normative change in mediating intervention effects on practices in this scoping review. We do note, however, that there have been multiple assessment methods applied in this area. For example, several interventions in this review demonstrated how qualitative implementation research could be applied to capture the perspectives of change agents and community members who influence adherence to norms and can provide important insights about how norms-shifting approaches are viewed [38,[54], [55], [56],^59^]. Three recent studies used randomized designs to compare the effect of norms-responsive interventions with more traditional educational approaches or a control group [37,47,52]. To understand the effectiveness of normative interventions, research designs that permit direct comparisons are valuable. However, such designs may not be feasible when programs are implemented at scale nor able to assess the sustainability of norms-shifting interventions over time. Alternatively, careful process evaluation and assessment of intermediate outcomes (including normative beliefs), implementation, and coverage can help assess pathways of impact [63].

### Limitations

This review focused on peer-reviewed literature, so relevant intervention approaches in grey literature may have been missed. We focused on intervention approaches aiming to reduce women’s undernutrition by improving quality and quantity of women’s diets, thus excluding articles on the design or implementation of interventions solely aimed at reducing overweight and obesity. We also limited our search to LMICs, potentially overlooking relevant normative approaches used in high-income settings.

### Implications for research and programming

Although attention to norms has increased over the past decade, and the interventions identified in this review appear to be promising based on the reported results, there is a need to better understand and measure the influence of normative programs on desired outcomes. Future research and programs that address norms can focus on filling key knowledge gaps. These include understanding the multiple pathways of influence betweem social and gender norms and women’s diets and the effect of different intervention approaches on these pathways. Knowledge development in this area would be facilitated by a comprehensive theoretical model, impact pathway, and/or theory of change that considers social norms as one set of influences within the larger array of factors to consider when designing public health interventions [64,65]. To be relevant across diverse contexts, future research could include multiple forms of malnutrition such as overweight and obesity, which are likely influenced by different pathways than those that lead to undernutrition. Additionally, there is also a need to develop valid and accurate measurements of norms and to assess how normative beliefs and related intermediate outcomes influence women’s diets [25,66,67].

We did not identify any interventions that used social media, radio, or television to address norms that influence women’s diets, which suggests an opportunity for future investigation. Finally, more attention is needed within nutrition-sensitive interventions to assess the impact women’s dietary outcomes, which could highlight additional benefits of these approaches and elucidate links among social and gender norms, empowerment, and women’s nutrition [68,69].

## Conclusions

Addressing influential social and gender norms is integral to creating household and community environments that enable the adoption of nutrition recommendations. The strategies summarized in this review provide a starting point for further implementation and evaluation of norms-focused and gender-transformative approaches, which hold promise for enhancing women’s nutritional outcomes. Ensuring that women consume healthy diets is critical to achieving nutrition goals and sustaining global food security; this requires moving beyond traditional SBC interventions that focus on knowledge and attitudes of individuals to also consider and respond to social and gender norms that influence relevant behaviors.

## Author contributions

The authors’ responsibilities were as follows – KLD, KL, LS: conceptualization; HCC, GWG, EP: search implementation; all authors: reviewing/extracting; KLD, GWG, KL, LS, EP: writing the manuscript; KL: led writing of the original manuscript; GWG: led the original review; EP: led updating of the review and revision of the manuscript; and all authors: read and approved the final manuscript.

## Conflict of interest

The authors report no conflicts of interest.

## Funding

The United States Agency for International Development provided financial support for this article through its flagship multisectoral nutrition project, Advancing Nutrition, a project of the United States Agency for International Development (USAID). It was prepared under the terms of contract 7200AA18C00070 awarded to JSI Research & Training Institute, Inc. The contents are the responsibility of JSI and do not necessarily reflect the views of USAID or the United States Government.

## References

[1] United Nations Sustainable Development Goals Overview (2023). https://unstats.un.org/sdgs/report/2019/goal-02/2022.

[2] Levels and trends in child malnutrition: UNICEF/WHO/World Bank group joint child malnutrition estimates: key findings of the 2023 edition (2023). https://www.who.int/publications/i/item/9789240073791.

[3] Gendered Health Analysis (2021). https://iris.paho.org/handle/10665.2/55432.

[4] Stevens G.A., Beal T., Mbuya M.N.N., Luo H., Neufeld L.M. (2022). Global Micronutrient Deficiencies Research Group, Micronutrient deficiencies among preschool-aged children and women of reproductive age worldwide: a pooled analysis of individual-level data from population-representative surveys. Lancet Glob. Health.

[5] Lander R.L., Hambidge K.M., Westcott J.E., Tejeda G., Diba T.S., Mastiholi S.C. (2019). Pregnant women in four low-middle income countries have a high prevalence of inadequate dietary intakes that are improved by dietary diversity. Nutrients.

[6] FAO, IFAD, UNICEF, WFP, and WHO (2023).

[7] UNICEF, World Bank, World Health Organization (2018). https://www.who.int/publications/i/item/9789241514064.

[8] Martin S.L., McCann J.K., Gascoigne E., Allotey D., Fundira D., Dickin K.L. (2020). Mixed-methods systematic review of behavioral interventions in low- and middle-income countries to increase family support for maternal, infant, and young child nutrition during the first 1000 days. Curr. Dev. Nutr..

[9] Martin S.L., McCann J.K., Gascoigne E., Allotey D., Fundira D., Dickin K.L. (2021). Engaging family members in maternal, infant and young child nutrition activities in low- and middle-income countries: a systematic scoping review, Matern. Child Nutr..

[10] Van den Bold M., Quisumbing A.R., Gillespie S. (2013). http://ebrary.ifpri.org/cdm/ref/collection/p15738coll2/id/127840.

[11] Kavle J.A., Landry M. (2018). Addressing barriers to maternal nutrition in low- and middle-income countries: a review of the evidence and programme implications. Matern. Child Nutr..

[12] Stefanik L., Hwang T. (2017). http://gender.careinternationalwikis.org/_media/care-social-norms-paper-web-final_july_2017.pdf.

[13] Cislaghi B., Heise L. (2019). Using social norms theory for health promotion in low-income countries. Health Promot. Int..

[14] Mackie G., Moneti F. (2014). What are social norms? How are they measured?. UNICEF and UCSD Center on Global Justice.

[15] Nguyen P.H., Frongillo E.A., Kim S.S., Zongrone A.A., Jilani A., Tran L.M. (2019). Information diffusion and social norms are associated with infant and young child feeding practices in Bangladesh. J. Nutr..

[16] Dickin K.L., Litvin K., McCann J.K., Coleman F.M. (2021). Exploring the influence of social norms on complementary feeding: a scoping review of observational, intervention, and effectiveness studies. Curr. Dev. Nutr..

[17] Yaker R. (2017). https://irh.org/resource-library/identifying-and-describing-approaches-and-attributes-of-normative-change-interventions-background-paper/.

[18] Institute for Reproductive Health (2021). https://www.irh.org/resource-library/social-norms-lexicon/.

[19] Legros S., Cislaghi B. (2020). Mapping the social-norms literature: an overview of reviews. Perspect. Psychol. Sci..

[20] Onah M.N., Horton S., Hoddinott J. (2021). What empowerment indicators are important for food consumption for women? Evidence from 5 sub-Sahara African countries. PLoS One.

[21] Gresham E., Bisquera A., Byles J.E., Hure A.J. (2016). Effects of dietary interventions on pregnancy outcomes: a systematic review and meta-analysis, Matern. Child Nutr..

[22] Victora C.G., Christian P., Vidaletti L.P., Gatica-Domínguez G., Menon P., Black R.E. (2021). Revisiting maternal and child undernutrition in low-income and middle-income countries: variable progress towards an unfinished agenda. Lancet.

[23] Esterik P.V. (1999). Right to food; right to feed; right to be fed. The intersection of women’s rights and the right to food. Agric. Hum. Values.

[24] Fox E.L., Davis C., Downs S.M., Schultink W., Fanzo J. (2019). Who is the woman in women’s nutrition? A narrative review of evidence and actions to support women’s nutrition throughout life. Curr. Dev. Nutr..

[25] The PASSAGES Project (2021). https://pdf.usaid.gov/pdf_docs/PA00ZPPJ.pdf.

[26] Keats E.C., Das J.K., Salam R.A., Lassi Z.S., Imdad A., Black R.E. (2021). Effective interventions to address maternal and child malnutrition: an update of the evidence, Lancet Child Adolesc. Health.

[27] Peters M.D., Godfrey C., McInerney P., Tricco A., Khalil H. (2020).

[28] Tricco A.C., Lillie E., Zarin W., O’Brien K.K., Colquhoun H., Levac D. (2018). PRISMA Extension for Scoping Reviews (PRISMA-ScR): checklist and explanation. Ann. Intern. Med..

[29] World Bank. World Bank Country and Lending Groups – World Bank Data Help Desk [Internet]. [cited 12 July, 2021]. Available from: https://datahelpdesk.worldbank.org/knowledgebase/articles/906519-world-bank-country-and-lending-groups.

[30] The Social Norms Learning Collaborative (2021). https://www.alignplatform.org/sites/default/files/2021-05/social-norms-atlas_final_v2.pdf.

[31] Covidence systematic review software [Internet]. Melbourne, Australia: Veritas Health Innovation. Available from: https://www.covidence.org.

[32] Alam A., Chowdhury M., Dibley M.J., Raynes-Greenow C. (2020). How can we improve the consumption of a nutritionally balanced maternal diet in rural Bangladesh? The key elements of the “balanced plate” intervention. Int J Environ Res Public Health.

[33] Aubel J., Touré I., Diagne M. (2004). Senegalese grandmothers promote improved maternal and child nutrition practices: the guardians of tradition are not averse to change. Soc. Sci. Med..

[34] Bao W., Ma A., Mao L., Lai J., Xiao M., Sun G. (2010). Diet and lifestyle interventions in postpartum women in China: study design and rationale of a multicenter randomized controlled trial. BMC Public Health.

[35] Cole D.C., Levin C., Loechl C., Thiele G., Grant F., Girard A.W. (2016). Planning an integrated agriculture and health program and designing its evaluation: experience from Western Kenya. Eval. Program. Plann..

[36] Entsieh A.A., Emmelin M., Pettersson K.O. (2015). Learning the ABCs of pregnancy and newborn care through mobile technology. Glob. Health Action.

[37] Galvin L., Verissimo C.K., Ambikapathi R., Gunaratna N.S., Rudnicka P., Sunseri A. (2023). Effects of engaging fathers and bundling nutrition and parenting interventions on household gender equality and women’s empowerment in rural Tanzania: results from EFFECTS, a five-arm cluster-randomized controlled trial. Soc. Sci. Med..

[38] Kadiyala S., Morgan E.H., Cyriac S., Margolies A., Roopnaraine T. (2016). Adapting agriculture platforms for nutrition: a case study of a participatory, video-based agricultural extension platform in India. PLOS ONE.

[39] Jeihooni A.K., Rakhshani T., Khiyali Z., Ebrahimi M.M., Harsini P.A. (2022). The effect of educational intervention based on theory of planned behavior on behavioral responses of premenopausal women in prevention of osteoporosis. BMC Womens Health.

[40] Liu N., Mao L., Sun X., Liu L., Yao P., Chen B. (2009). The effect of health and nutrition education intervention on women’s postpartum beliefs and practices: a randomized controlled trial. BMC Public Health.

[41] Morrison J., Giri R., Arjyal A., Kharel C., Harris-Fry H., James P. (2021). Addressing anaemia in pregnancy in rural plains Nepal: a qualitative, formative study, Matern. Child Nutr..

[42] Nandi S., Schneider H. (2014). Addressing the social determinants of health: a case study from the Mitanin (community health worker) programme in India. Health Policy Plan.

[43] Nguyen P.H., Sanghvi T., Kim S.S., Tran L.M., Afsana K., Mahmud Z. (2017). Factors influencing maternal nutrition practices in a large scale maternal, newborn and child health program in Bangladesh. PLOS ONE.

[44] Ochieng J., Afari-Sefa V., Karanja D., Kessy R., Rajendran S., Samali S. (2018). How promoting consumption of traditional African vegetables affects household nutrition security in Tanzania. Renew. Agric. Food Syst..

[45] Shivalli S., Srivastava R.K., Singh G.P. (2015). Trials of improved practices (TIPs) to enhance the dietary and iron-folate intake during pregnancy- a quasi experimental study among rural pregnant women of Varanasi, India. PLOS ONE.

[46] Yilma H., Sedlander E., Rimal R.N., Pant I., Munjral A., Mohanty S. (2020). The reduction in anemia through normative innovations (RANI) project: study protocol for a cluster randomized controlled trial in Odisha, India. BMC Public Health.

[47] Talegawkar S.A., Jin Y., Sedlander E., Ganjoo R., Behera S., DiPietro L. (2021). A social norms-based intervention improves dietary diversity among women in rural India: the Reduction in Anemia through Normative Innovations (RANI) Project. Nutrients.

[48] MacDonald C.A., Aubel J., Aidam B.A., Girard A.W. (2020). Grandmothers as change agents: developing a culturally appropriate program to improve maternal and child nutrition in Sierra Leone. Curr. Dev. Nutr..

[49] Dykes F., Lhussier M., Bangash S., Zaman M., Lowe N. (2012). Exploring and optimising maternal and infant nutrition in North West Pakistan. Midwifery.

[50] Isler J., Sawadogo N.H., Harling G., Bärnighausen T., Adam M., Sié A. (2020). ‘If he sees it with his own eyes, he will understand’: how gender informed the content and delivery of a maternal nutrition intervention in Burkina Faso. Health Policy Plan.

[51] Nguyen P.H., Frongillo E.A., Sanghvi T., Wable G., Mahmud Z., Tran L.M. (2018). Engagement of husbands in a maternal nutrition program substantially contributed to greater intake of micronutrient supplements and dietary diversity during pregnancy: results of a cluster-randomized program evaluation in Bangladesh. J. Nutr..

[52] Prost A., Harris-Fry H., Mohanty S., Parida M., Krishnan S., Fivian E. (2022). Understanding the effects of nutrition-sensitive agriculture interventions with participatory videos and women’s group meetings on maternal and child nutrition in rural Odisha, India: a mixed-methods process evaluation, Matern. Child Nutr..

[53] Ragasa C., Lambrecht I., Mahrt K., Zhao H., Aung Z.W., Scott J. (2021). Can nutrition education mitigate the impacts of COVID-19 on dietary quality? Cluster-randomised controlled trial evidence in Myanmar’s Central Dry Zone, Matern. Child Nutr..

[54] Ridolfi R., Stormer A., Mundy G. (2019). Transforming data into action – implementing gender analyses in nutrition-sensitive agriculture interventions: an experience from Cambodia. Adv. Gend. Res..

[55] van den Bold N., Dillon A., Olney D., Ouedraogo M., Pedehombga A., Quisumbing A. (2015). Can integrated agriculture-nutrition programmes change gender norms on land and asset ownership? Evidence from Burkina Faso. J. Dev. Stud..

[56] Diamond-Smith N., Mitchell A., Cornell A., Dahal M., Gopalakrishnan L., Johnson M. (2022). The development and feasibility of a group-based household-level intervention to improve preconception nutrition in Nawalparasi district of Nepal. BMC Public Health.

[57] Katenga-Kaunda L.Z., Iversen P.O., Holmboe-Ottesen G., Fjeld H., Mdala I., Kamudoni P.R. (2020). Dietary intake and processes of behaviour change in a nutrition education intervention for pregnant women in rural Malawi: a cluster-randomised controlled trial. Public Health Nutr.

[58] Khani Jeihooni A., Jormand H., Saadat N., Hatami M., Abdul Manaf R., Afzali Harsini P. (2021). The application of the theory of planned behavior to nutritional behaviors related to cardiovascular disease among the women. BMC Cardiovasc. Disord..

[59] Wable Grandner G., Rasmussen K.M., Dickin K.L., Menon P., Yeh T., Hoddinott J. (2022). Storytelling for persuasion: insights from community health workers on how they engage family members to improve adoption of recommended maternal nutrition and breastfeeding behaviours in rural Bangladesh. Matern. Child Nutr..

[60] Schuster R.C., Szpak M., Klein E., Sklar K., Dickin K.L. (2019). “I try, I do”: child feeding practices of motivated, low-income parents reflect trade-offs between psychosocial- and nutrition-oriented goals. Appetite.

[61] Sraboni E., Quisumbing A. (2018). Women’s empowerment in agriculture and dietary quality across the life course: evidence from Bangladesh. Food Policy.

[62] Lentz E.C. (2018). Complicating narratives of women’s food and nutrition insecurity: domestic violence in rural Bangladesh. World Dev.

[63] Rawat R., Nguyen P.H., Ali D., Saha K., Alayon S., Kim S.S. (2013). Learning how programs achieve their impact: embedding theory-driven process evaluation and other program learning mechanisms in Alive & Thrive. Food Nutr. Bull..

[64] UNICEF (2021). UNICEF Conceptual Framework on Maternal and Child Nutrition.

[65] Bronfenbrenner U. (1977). Toward an experimental ecology of human development. Am. Psychol..

[66] Davin C. (2019). Resources for measuring social norms: a practical guide for programme implementers. Learning Collaborative to Advance Normative Change. Align Platform.

[67] Costenbader E., Cislaghi B., Clark C.J., Hinson L., Lenzi R., McCarraher D.R. (2019). Social norms measurement: catching up with programs and moving the field forward. J. Adolesc. Health.

[68] Feed the Future. Gender (2020). https://basis.ucdavis.edu/project/gender-nutrition-sensitive-agricultural-programs-and-resilience-bangladesh.

[69] Harris-Fry H.A., Paudel P., Harrisson T., Shrestha N., Jha S., Beard B.J. (2018). Participatory women’s groups with cash transfers can increase dietary diversity and micronutrient adequacy during pregnancy, whereas women’s groups with food transfers can increase equity in intrahousehold energy allocation. J. Nutr..

