# Supplementary material for: How Do Social and Behavioral Change Interventions Respond to Social Norms to Improve Women’s Diets in Low- and Middle-Income Countries? A Scoping Review
Source: Curr Dev Nutr. 2024 May 11;8(6):103772. doi: 10.1016/j.cdnut.2024.103772 (PMC11214384; doi:10.1016/j.cdnut.2024.103772)
Supplement: Multimedia component 1 [file mmc1.docx]

**Supplement 1: Search Terms Used in PubMed**

| **Search domains** | **Search terms** |
| --- | --- |
| 1. Women’s diets | (women OR woman OR mother* OR WRA OR childbear* OR pre-conception OR preconception OR maternal OR pregnan* OR lactating OR breastfeeding OR nutrition* OR diet* OR food* OR intake* OR hunger OR hungry OR meal* OR consumption OR MUAC OR “mid upper arm circumference” OR underweight OR undernutrition OR undernourished OR malnourished OR nutrient* OR anemi* OR bmi OR “body mass index” OR “gestational weight gain” OR “maternal stature”) |
| 2. Social norms | “social norms”[MeSH] OR "Social Conformity/psychology"[MeSH] OR norm OR norms OR normative OR cultur* OR tradition* OR stereotype* OR perception* OR ideal* OR taboo* OR “gender expectation*” OR “gendered expectation*” OR “gender role*” OR “gender dynamic*” OR patriarch* OR matriarch* OR “family structure*” OR “family role*” OR “family dynamic*” OR “family relationship*” OR “family hierarchy” OR “change agent*” OR “reference group*” OR “powerful group*” OR “social influence” OR “gatekeeper” OR “food prescription*” OR “food proscription*” OR “food avoidance*” OR “food restriction*” OR “food belief*” OR “food habit*” OR “food choice*” OR “food custom*” OR “body image” OR “body satisfaction” OR “eating down” OR sacrific* OR self-interest OR “self interest” OR selflessness OR self-image OR “self image” OR lazy OR laziness |
| 3. Interventions | intervention* OR program* OR messag* OR SBC OR SBCC OR “social change” OR “behavioral change” OR “behavioural change” OR “behavior change” OR “behaviour change” OR “change behavior*” OR “change behaviour*” OR “nutrition education” OR counseling OR counselling OR “interpersonal communication” OR “peer support” OR “support group” OR “discussion group” OR “peer educat*” OR “health educat*” OR “health promotion” OR media OR “mHealth” OR “community mobilization” OR “community engagement” OR outreach OR “social mobilization” OR “norms shifting” |
| 4. LMIC Context | “Developing country” OR “Developing countries” OR “low-income countries” OR “low-income country” OR “lower middle-income country” OR “lower middle-income countries” OR afghan* OR albania* OR algeria* OR angola* OR “antigua and barbuda” OR armenia* OR azerbaijan* OR argentin* OR bangladesh* OR belarus* OR belize* OR benin* OR Bhutan* OR Bolivia* OR “Bosnia and Herzegovina*” OR Botswan* OR Brazil* OR “Burkina Faso” OR Burundi* OR “Cabo Verde” OR “Cape Verde” OR Cambodia* OR Kampuchea* OR Cameroon* OR “Central African Republic” OR chad* OR chile* OR china OR chinese OR colombia* OR comoros* OR congo* OR zaire OR “costa rica*” OR “Côte d'Ivoire” OR “Cote d'Ivoire” OR “Ivory Coast” OR Croatia* OR cuba* OR Djibouti* OR dominica* OR Ecuador* OR Egypt* OR “El Salvador*” OR Eritrea* OR Ethiopia* OR fiji* OR Gabon* OR gambia* OR Georgia* OR Ghana* OR grenada* OR Guatemala* OR guinea* OR Guyana* OR haiti* OR hondura* OR Hungar* OR india* OR Indonesia* OR iran* OR Iraq* OR Jamaica* OR Jordan* OR Kazakh* OR Kenya* OR Kiribati* OR korea* OR Kosovo* OR kyrgyz* OR “Lao PDR” OR laos OR laotian OR Latvia* OR leban* OR lesotho OR basotho OR mosotho OR Liberia* OR Libya* OR lithuania* OR madagascar OR malagasy OR Malawi* OR malays* OR Maldives OR mali* OR “Marshall Islands” OR Mauritania* OR Mauritius OR mexic* OR micronesia* OR moldova* OR Mongolia* OR Montenegro OR morocc* OR Mozambi* OR myanmar OR burma* OR burmese OR Namibia* OR Nauru* OR nepal* OR “North Macedonia” OR nicaragua* OR niger* OR Nigeria* OR palau* OR Pakistan* OR panama* OR paraguay* OR peru* OR philippines OR phillippines OR philipines OR phillipines OR filipino* OR Poland OR Polish OR Portug* OR Romania* OR Russia* OR Rwanda* OR ruanda* OR samoa* OR “São Tomé and Principe” OR “sao tome and Principe” OR Senegal* OR “Sierra Leone” OR “Solomon Islands” OR somali* OR “south africa*” OR “Sri Lanka*” OR “Saint Vincent and the Grenadines”[MeSH] OR sudan* OR suriname OR swazi* OR eswatini* OR Syria* OR tajik* OR Tanzania* OR Thai* OR togo* OR tonga* OR Tunisia* OR turk* OR Uganda* OR ukrain* OR Uruguay* OR uzbek* OR Vanuatu* OR “West Bank and Gaza” OR Venezuela* OR Vietnam* OR yemen* OR Yugoslavia* OR Zambia* OR Zimbabwe* |
| Final search | #1 AND #2 AND #3 AND #4 |

Note: all terms search as title/abstract/keyword unless otherwise noted; Search results limited to English language, full text papers published between January 1, 1990 and August 31, 2023 with available abstracts.
